# Community Attitudes Reflect Reporting Rates and Prevalence of Animal Mistreatment

**DOI:** 10.3389/fvets.2021.666727

**Published:** 2021-10-28

**Authors:** Carmen Glanville, Jennifer Ford, Rebecca Cook, Grahame J. Coleman

**Affiliations:** ^1^Animal Welfare Science Centre, Faculty of Veterinary and Agricultural Sciences, University of Melbourne, Melbourne, VIC, Australia; ^2^JCFord Consultancy, Melbourne, VIC, Australia; ^3^RSPCA Victoria, Melbourne, VIC, Australia

**Keywords:** animal mistreatment, animal welfare, animal cruelty, attitudes, community attitudes, interventions, prevention

## Abstract

Community attitudes toward the treatment of animals are important to understand for the development of intervention programs to prevent mistreatment. We aimed to investigate whether previously identified differences between local government areas (LGAs) in the rates of animal mistreatment reporting and prevalence were reflected by differences in community attitudes. In addition, attitudinal differences based on target species (dogs, cats, horses) and participant gender were considered. A representative telephone survey (*N* = 1,801) was conducted across six LGAs. Attitudinal themes included affection toward animals, valuing of animals, attitudes toward caring for own animals, and concern for the mistreatment of other animals. Factorial ANOVA was used to identify differences between high and low reporting LGAs, region types (regional, interface, metropolitan), and target species (cat, dog, horse). Respondents from high reporting LGAs demonstrated slightly more affection for animals *F*_(1,1679)_ = 19.401, *p* < 0.001, ${\text{ω}}_{\text{p}}^{2}$ = 0.011 and stronger subjective norms *F*_(1,999)_ = 16.31, *p* < 0.001, ${\text{ω}}_{\text{p}}^{2}$ = 0.015 than those from low reporting LGAs, but did not differ on the other variables. Participants in areas of high prevalence (regional areas) did not display lower levels of affection or concern for the mistreatment of animals as a whole, nor did they value animals less. However, regional differences were found for several items regarding caring for one's own animals: two behavioral beliefs and two control beliefs. Additionally various differences were found between the regions regarding the level of concern for mistreatment when broken down into the different species. Gender effects were also common. While the attitudinal results do reflect animal mistreatment prevalence and reporting rates, they also highlight the complexity of community attitudes. As such, interventions to prevent mistreatment must have clear targets including the audience, behavior, and species. Targeting smaller regions and thoroughly investigating their unique perspectives, challenges, and strengths are likely to be more effective than generic campaigns.

## Introduction

Animal mistreatment takes many forms. Whether, it be intentional abuse or unintentional neglect, the harms to the animal can be significant and enduring (1, 2). However, other than what can be gleaned from voluntary community reports of mistreatment, little is known about the prevalence or nature of animal mistreatment in the community. Having a sound understanding of the problem and its drivers is key to prevention and intervention development, which is a growing field of applied scholarship within the animal welfare domain (3, 4). This paper is the second from a large, representative telephone survey that aims to contribute to this knowledge gap by using principles of social psychology to better understand animal mistreatment in Victoria, Australia.

This research was prompted by observations that different local government areas (LGAs) in Victoria had differing levels of community reporting to the Royal Society for the Prevention of Cruelty to Animals Victoria (RSPCA Vic, the primary enforcement agency regarding animal mistreatment). Some LGAs had consistently high rates of animal mistreatment reports while others had consistently low rates of reports. These trends have also been identified in other states of Australia and internationally, sparking considerable media attention around so-called “cruelty hotspots” (5–8). However, in our first paper, we demonstrated that areas with high rates of community reports to RSPCA Victoria did *not* have a higher prevalence of mistreatment, simply a greater propensity to report it (9). Additionally attitudes toward reporting specifically (based on Theory of Planned Behavior constructs) were poor predictors of reporting behavior. Hence, we questioned whether people in high reporting areas have more positive attitudes toward animals and their treatment and therefore, are more likely to report when they see something wrong. This became the first research question to be addressed in this paper.

Where we did identify a difference in prevalence, it was between region types. We compared the three main region types in Victoria according to Local Government Comparator Groups: metropolitan (within the capital city of Melbourne), interface (peri-urban), and regional cities (smaller cities outside of Melbourne) (10). Participants in regional LGAs reported having witnessed 2.5–3 times more separate incidents of mistreatment than participants in metropolitan and interface areas. As such, we questioned whether people in areas with higher prevalence of mistreatment (regional areas) had correspondingly more negative attitudes toward animals and their treatment. This became the second research question to be addressed here.

**Figure 1:**
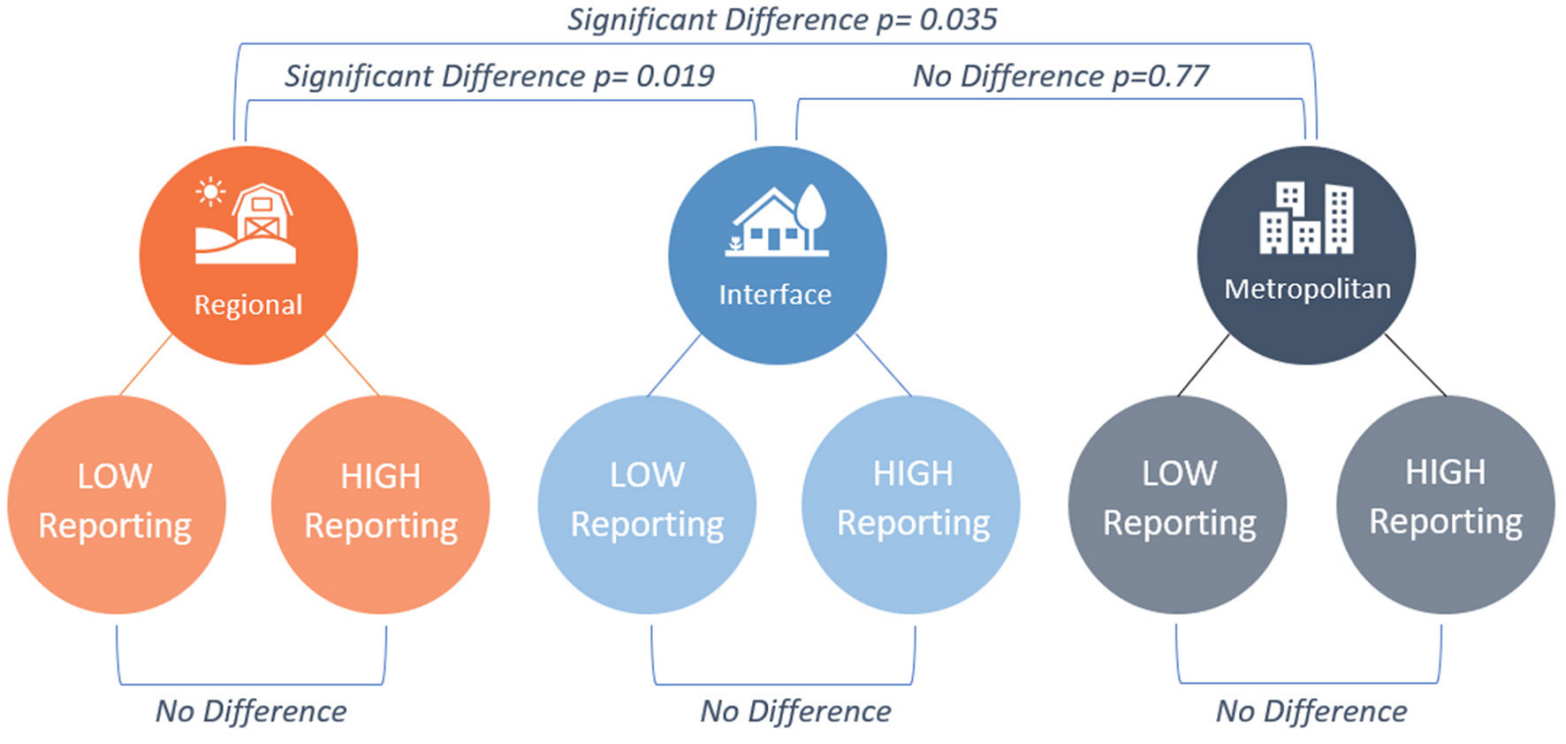
Visual representation of animal mistreatment prevalence results from the first paper of the present study (9). Six Local Government Areas were surveyed using a factorial design; region type × mistreatment reporting rate. Factorial ANOVA identified no difference in prevalence between matched pairs of LGAs, but a significant difference between region types [omnibus test: *F*_(2,444)_ = 3.554, *p* = 0.029; *post-hoc* pairwise comparison *p*-values provided in figure].

A graphical summary of the prevalence results from Glanville et al. (9) pertinent to the current paper is provided in Figure 1.

So what are attitudes and why do they matter in the study of animal mistreatment? Attitudes are evaluations of an attitude object (anything one can hold an attitude about; person, animal, event, behavior) based on the beliefs a person holds about that object and how those beliefs align with and serve to achieve value-based goals (11). Along with associated knowledge and values, attitudes are a behavioral expression (that is, a verbal behavior) that can be used to monitor trends in population characteristics. However, attitudes are also important because, at an individual level, they can act as the prime determinants of volitional human behaviors, that is, those behaviors that are carried out as a conscious choice (12). Attitudes have long been recognized as important drivers of human behavior (13). Community attitudes (the attitudes of people in a given community) are often a focus of research and interventions for social issues. The implicit reasoning behind this, which is often not articulated, is that community attitudes may reflect the underlying social problem and influence behaviors associated with that problem. In this way, they are sometimes used as a “yard stick” or proxy measure for an underlying social issue when direct measurement is difficult such as in child abuse, racism, substance abuse, family violence, or ableism [e.g., (14–16)]. Understanding community attitudes can also assist in the development of community-level intervention and prevention programs in two ways. First, changing attitudes as part of a community intervention can lead to changes in the relevant behaviors. Second, measuring attitudes over time can help track the impact of various events, including interventions. Furthermore community attitudes are especially important for the study of animal mistreatment given that the current intervention and enforcement model relies on public reports as a first step (17). Consequently, the community's attitudes toward animals and their treatment play a vital role in protecting animals.

While there has been significant work on community attitudes toward the treatment of livestock (18–21) and various other animal-related regulation or policy issues (22, 23), little empirical work has examined community attitudes toward the treatment and mistreatment of companion species; those most commonly reported by the community for mistreatment. In a systematic review of farm animal welfare attitudinal research, Clark et al. (21) identified regional differences in people's concern for some aspects of farm animal welfare. Given these findings, regional differences could be present in people's attitudes toward companion animal species. To date, attitudinal studies regarding companion animal species have mainly focused on specific management behaviors and drivers of these at the individual level as opposed to community-level trends [e.g., (24–27)]. With regards to mistreatment specifically, there has similarly been attitudinal research conducted at the individual level, primarily focused on intentional cruelty (28, 29), although little work has been conducted at the community level. In addition, many of the aforementioned attitudinal studies rely on convenience samples. While such studies can be useful in examining drivers of individual behavior, they cannot give an accurate representation of community-level attitudes and trends. As prevention programs are often targeted at the community level, it is important to investigate these community-level attitudes.

But what attitudes are relevant? Given the dual role of community members as both potential performers and witnesses/reporters of mistreatment, their attitudes toward both their own animals and others are important. The current literature on individual attitudes toward animals provides some potentially useful dimensions for exploration. How much a person likes or dislikes the animal has been found to relate to other attitudes around pet ownership (30); therefore, affection toward animals is potentially important. In Victoria, there are clear differences in the representation of different species of animals in animal mistreatment reports. Dogs are the most commonly reported species, followed by horses, and finally cats (31). Differences in attitudes and affection toward various species are well-documented in the literature [e.g., (32, 33)] and as such, the target species is likely to be influential. Henry (34) found that exposure to animal mistreatment was related to decreased sensitivity or concern for the mistreatment of animals. As such, we could predict that people in areas of high rates of mistreatment (and therefore, more likely to witness mistreatment) would have less concern for the mistreatment of animals. Finally, it is logical that attitudes toward caring for one's own animal would be important in understanding how a community treats their animals. Such attitudes toward management behaviors are often investigated using the Theory of Planned Behavior as a conceptual framework [e.g., (35–38)].

Therefore, the present study aimed to determine whether differences in reporting rates and prevalence of mistreatment were reflected in differences in community attitudes. Three specific research questions were tested:

1) Do respondents in areas of high *reporting* have more positive attitudes toward animals?
2) Do respondents in areas of high *prevalence* have more negative attitudes toward animals?
3) Do attitudes differ for different target species?

## Materials and Methods

### Human Ethics Approval

This project was conducted in accordance with the National Statement on Ethical Conduct in Human Research (2007) guidelines and regulations. The University of Melbourne Veterinary and Agricultural Sciences Human Ethics Advisory Group granted approval for both the preliminary focus groups (ID: 1853397) and survey (ID: 1954263). Prior to partaking, all participants provided informed verbal consent, were advised they could end their participation at any time, and were welcome to withdraw their data on completion of the task.

### Focus Groups

Best practice questionnaire development combines deductive methods (literature review) and inductive methods (focus groups, interviews) for item generation (39). As such, exploratory focus groups were used to elicit key attitudinal themes and individual items from the target audience for the subsequent Computer Assisted Telephone Interview (CATI) questionnaire. Focus groups were used over individual interviews as they require less time to gain a broader range of views and can provide additional insights given the interaction between participants (e.g., reactions to opposing views). Four sessions were conducted at community sites; a library and a neighborhood house. The sessions were held in the City of Latrobe in Victoria, Australia as this was the focal site for a separate project conducted by our industry partners, providing the opportunity to integrate this work. Recruitment occurred through non–animal related social media groups (buy/swap/sell groups and community noticeboard groups) and a financial incentive (AU$60) was provided to attract a wide audience. A total of 37 adult individuals participated across four focus groups; 26 female, 10 male, and 1 gender non-binary, aged from 18 to 65 years. No recruits were excluded. A structured protocol covered themes of general attitudes toward animals and their care, what constitutes mistreatment, and potential drivers of mistreatment.

Common themes and beliefs were identified for inclusion in the questionnaire. The most pertinent of these was that people who mistreat animals do not value them as much as others. Examples given for this were people giving up their animals if they were sick or impounded and obtaining new animals instead of paying for the associated costs.

### Survey Questionnaire

Given the themes identified in the focus groups and literature review, the attitudinal elements of the survey included (1) affection toward animals (cats, dogs, horses), (2) valuing animals, (3) attitudes toward caring for animals, and (4) level of concern for different types of mistreatment (Table 1). Demographic variables (age, education, country of birth, income bracket) were also included to fulfill sampling quotas and to allow for *post-hoc* weighting of the data (see section Sampling and Questionnaire Delivery).

**Table 1:** Questionnaire items used in Computer Assisted Telephone Interview.

**Topic**	**Items**	**Response scale**
1. Affection	*Now using a scale of 0 to 10 where 0 is Hate, 10 is Love, and 5 is Neutral (neither like or dislike), how do you feel about…* a) Cats b) Dogs c) Horses	0–10
2. Valuing	*To what extent do you agree or disagree that…* VAL1 Animals are a valuable part of our society VAL2 My animals make my life better VAL3 If my animal were to develop an unexpected illness or injury, I would prefer to get a new animal than pay for expensive vet fees VAL4 I would prefer to get a new animal than to pay reclaim fees if my animal ended up at a pound or shelter VAL5 It is not worth the money to take my animal to the vet for general health care when it's not sick or injured	1. Strongly disagree 2. Disagree 3. Neither agree nor disagree 4. Agree 5. Strongly agree
3. Caring for animals	*To what extent do you agree or disagree that…* PBC1 I don't have time to look after my animals the way I would like PBC2 I'm unsure how best to care for my animals PBC3 I can't afford to look after my animals how I would like SN1 People who are important to me would expect me to provide a high level of care for my animals SN2 It matters to me what people think about how I treat my animals SN3 People who are important to me wouldn't care how I looked after my animals BB1 How my animal feels, whether they are happy and healthy, is important to me BB2 My animal's health and happiness depend on how I look after them BB3 Providing a high level of care for my animal/s is important to me	1. Strongly disagree 2. Disagree 3. Neither agree nor disagree 4. Agree 5. Strongly agree
4. Concern for mistreatment	*How concerned would you be about…?* Species-specific attitude items A cat that consistently has fleas Someone drowning unwanted kittens A horse with overgrown hooves A horse that is attended to once a week A dog that consistently has fleas A dog that is consistently tied up for most of the day All species items: each participant only asked about one species A cat (dog or horse) that is underweight, such that you can see its ribs or hip bones A cat (dog or horse) that is on its own and receives little attention A cat (dog or horse) kept outside (horse—in a paddock) without shelter A cat (dog or horse) that is clearly injured or ill, but not receiving veterinary treatment Someone intentionally hurting a cat (dog or horse) other than for training Someone intentionally hurting a cat (dog or horse) for the purposes of training or to teach it a lesson (e.g., hitting)	0) Not concerned at all 1) 2) 3) 4) 5) 6) 7) 8) 9) 10) Extremely concerned

Affection for the species was measured in a similar way to Toukhsati et al. (40), although instead of a 7-point scale, we used a 10-point scale to provide greater variation/sensitivity. Valuing animals items were taken directly from views expressed by focus group participants. Attitudes toward caring for pets were modeled on the Theory of Planned Behavior, one of the most widely used and empirically tested models of volitional behavior. The Theory of Planned Behavior uses three key attitudinal elements to predict behavioral intention and behavior itself: attitudes toward the behavior (evaluation of the outcomes of the behavior), subjective norms (perceived social pressure), and perceived behavioral control (11). As attitudes are difficult to assess directly, these elements are typically measured through salient belief statements. As such, to assess attitudes toward caring for pets, items reflecting behavioral beliefs, normative beliefs, and control beliefs that were expressed by focus groups participants were included. Concern for the mistreatment of animals items were derived from the Attitudes towards the Treatment of Animals Scale (28) while being modified to reflect common mistreatment complaints within Victoria for each species (dog, cat, horse). Eight concern items were derived for each species, six of which were identical for each species while two items were species specific. Due to the length of the survey, each participant only answered “concern for mistreatment” questions for one species to avoid questionnaire fatigue and drop out; pre-testing identified that completing all three species was tiresome for participants. Participants were randomly allocated to the different species, with 100 people for each species in each LGA (600 total for each species).

### Sampling and Questionnaire Delivery

The attitudinal items discussed here were part of a larger survey with the full methodology and Local Government Area (LGA) selection process outlined in Glanville et al. (9). In short, sampling followed a 2×3 factorial design based on (1) the rate of reports made to RSPCA Victoria (high reporting vs. low reporting) and (2) region type (regional, interface, metropolitan) (Table 2). High and low reporting pairs within each region type were matched based on socioeconomic index and population to limit the impact of these factors.

**Table 2:** Sampling design and local government areas selected.

		**Region type**		
		**Regional**	**Interface**	**Metro**
Report rate	High	Latrobe	Yarra ranges	Melbourne
	Low	Mildura	Wyndham	Stonnington

The questionnaire was delivered as a CATI by the Social Research Center (Australian National University). Commercial telephone lists were used for Random Digit Dialing with a mix of 80% mobile numbers and 20% landlines. Sampling quotas were set for gender and age for each LGA based on the latest census benchmarks (2016). Additional *post-hoc* weighting was calculated by the Social Research Center to improve the population representation of the sample. This *post-hoc* weighting included the computation of a design weight for each respondent as the inverse of their probability of selection. This design weight was then calibrated to match population benchmarks for key demographic factors including sex, education level, country of birth, volunteer status, and telephone status. Data collection occurred from April 29 to May 27, 2019. There was a 9.4% AAPOR Response Rate 3 (41) and a 19.5% overall cooperation rate, with variation between landline (31.8%) and mobile (18.1%) frames.

### Statistical Analyses

All data analyses were conducted using IBM SPSS Statistics for Windows, version 25 (IBM Corp., Armonk, NY, USA). Descriptive statistics were used to screen all variables prior to detailed analyses. Most of the variables showed significant skew, however, given the large sample size (total *N* = 1,801) and equal sample sizes between LGAs, this was not considered a problem for factorial analysis of variance (42, p. 307).

Factorial analysis of variance (ANOVA) was used to investigate the differences between high and low reporting LGAs and different region types (regional, interface, metropolitan) for all variables. Gender was included as an additional factor in all ANOVAs given the well-documented relationship between gender and attitudes toward animals [e.g., (42)]. Only two individuals in the sample identified as “other gender” and hence any conclusions drawn from these two people would not be representative. Consequently, these two cases were excluded from the analyses. Other demographic variables were not included in the ANOVAs as this would have resulted in small and unequal cell frequencies, thus violating the statistical assumptions of these tests. These variables were only collected to ensure the representativeness of the overall sample. Estimated marginal means (EMM) comparisons (least significant difference) were used to identify differences within significant effects where there were more than two groups. The Omega squared statistic (${\text{ω}}_{\text{p}}^{2}$) was calculated to report effect sizes (43).

#### Affection

Affection scores were analyzed for each individual species and as a whole. The average of the affection scores for the three different species was taken and subject to the same factorial ANOVAs.

#### Valuing Animals

Of the five items concerning valuing animals, one was applicable to all participants (*animals are a valuable part of society*), while the remaining four were only asked of participants who owned animals. Items did not sufficiently correlate (*r* < 0.3) with each other to perform dimension reduction techniques (e.g., principal components analysis). Hence, all items were examined individually.

#### Attitudes Toward Caring for Animals

These items only applied to participants that owned animals. Again, correlations between items were low and all items were analyzed individually, except for the subjective norm items. The subjective norm items were constructed using the value expectancy model of the Theory of Planned Behavior (44), which incorporates both the extent to which the individual believes important others would approve/disapprove of the behavior, and their motivation to comply with such expectations. A single subjective norm measure was calculated using SN1–SN3 as a measure of normative belief (subtracted because SN3 is reverse worded) and SN2 as a measure of motivation to comply (Table 1). This product was then divided by 3 (number of SN items) to convert it back to a 5-point scale for ease of interpretation alongside the other 5-point items. Thus, subjective norm was calculated as SN = [(SN1 – SN3) × SN2]/3.

While adding in an additional factor to account for the species of animals owned was trialed, this led to greatly varied cell sizes and heterogeneity of variance. Consequently, this was not used in the final analyses.

#### Concern for Mistreatment

To develop a single concern for mistreatment score for analysis, both exploratory factor analysis (principal axis factoring) and mean scale scores (mean of all items in the scale) were trialed on the different species sub-scales. One-factor solutions were found for each species and factor scores were saved using the regression method. Each species sub-scale demonstrated high internal reliability (Cronbach's alpha cat = 0.89, dog = 0.90, horse = 0.85) and there were high correlations between the factor scores and mean scale scores (*r* > 0.96). Therefore, the mean scale scores were used for subsequent analyses as they are easier to interpret for effect sizes and easier to replicate by others.

As two items for each of the species were unique (see Table 1), to ensure comparisons between species sub-scales were valid, the aforementioned analyses were conducted with both the full set of items and only those that were identical between species. Correlations between the reduced and full item sets were very high (*r* > 0.97) and hence the full item sets were used.

Because each participant was only asked concern for mistreatment items relating to one of the three species under investigation (cat, dog, horse), all responses were combined into a single concern for mistreatment variable with a second categorical variable created to indicate which species that individual had been assigned. Subsequently, analyses with these data were conducted using a three-way factorial ANOVA with the independent variables being (1) reporting rate (HR/LR), (2) region type (regional, interface, metropolitan), and (3) species (cat, dog, horse).

During initial data screening, significant correlations were found between affection scores and concern for mistreatment scores. Consequently, affection was included as a covariate in a second phase of analysis to determine whether concern for mistreatment was primarily based on whether people like these animals or whether acceptable treatment has a component distinct from affection.

## Results

### Participant Demographics

Overall, 1,801 individuals were surveyed; 300 from Latrobe, Yarra Ranges, Melbourne, Stonnington, and Wyndham, and 301 from Mildura. Participant ages ranged from 18 to 93 (*M* = 49.32, *SD* = 16.86) with 45.6% identifying as male, 53.7% female, 0.2% as other gender, and 0.4% undisclosed (0.1% discrepancy due to rounding).

#### Animal Owning Sub-sample

Several questionnaire items applied to animal owners only (Table 1). When the *post-hoc* weighting was applied, 1,114 (61.9%) participants owned an animal. The rate of ownership differed across the region types with 77% of the people sampled in regional areas owning an animal, 65.7% in interface, and 42.9% in metropolitan. Most owners only owned one (62.1%) or two (24.1%) category/species of animal.

### Affection for Animals

Overall, dogs were the most well-liked species (*M* = 8.90, *SD* = 1.80), followed by horses (*M* = 7.51, *SD* = 2.31), and finally cats (*M* = 6.97, *SD* = 2.86). Of all participants, 13.8% did not like cats (scored 4 or less) compared with 1.7% for dogs and 5.2% for horses, and 86 people (4.8%) said that they hated cats (scored 0).

For all species, the ANOVA model accounted for a very low amount of variance in affection: $R_{dog}^{2}$ = 0.038, $R_{cat}^{2}$= 0.035, $R_{horse}^{2}$= 0.022, and $R_{all}^{2}$= 0.034. While several statistically significant effects were found, most of the effect sizes were so small as to be of little, if any, practical significance. The largest effect was that of gender on affection for cats; females had more affection for cats than males *F*_(1,1662)_ = 35.51, *p* < 0.001, ${\text{ω}}_{\text{p}}^{2}$ = 0.020. Small differences were found between region types and reporting rates for dogs; metropolitan areas had less affection for dogs than regional and interface areas, and low reporting areas had less affection than high reporting areas. When averaged across the three species, females had higher levels of affection than males *F*_(1,1662)_ = 33.07, *p* < 0.001, ${\text{ω}}_{\text{p}}^{2}$ = 0.019 and people in high reporting areas had higher levels of affection than those in low reporting areas *F*_(1,1662)_ = 20.26, *p* < 0.001, ${\text{ω}}_{\text{p}}^{2}$ = 0.011.

### Valuing Animals

Again, while several statistically significant effects were found for valuing animals, the effect sizes suggest limited practical significance. The largest effects found were in value items 1 (*Animals are a valuable part of our society*) and 5 (*It is not worth the money to take my animal to the vet for general health care when it's not sick or injured*). In both of these items, females displayed a slightly higher sense of value than did males: Val1 *EMM*_female_ = 4.86, *SD*_female_ = 0.39, *EMM*_male_ = 4.74, *SD*_male_ = 0.59, *F*_(1,1662)_ = 27.08, *p* < 0.001, ${\text{ω}}_{\text{p}}^{2}$ = 0.015; Val5 *EMM*_female_ = 1.58, *EMM*_male_ = 1.93, *F*_(1,1008)_ = 24.12, *p* < 0.001, ${\text{ω}}_{\text{p}}^{2}$ = 0.022.

A small three-way interaction was found in value item 1 (*Animals are a valuable part of our society*), whereby males in the low reporting metropolitan region scored lower and had greater variability (*EMM* = 4.45, *SD* = 1.03) than males in all other groups (*EMM* = 4.78–4.81, *SD* = 0.40–0.50). Similarly, females in the low reporting regional area scored lower (*EMM* = 4.76, *SD* = 0.52) than females in all other groups (*EMM* = 4.86–4.90, *SD* = 0.30–0.49) (Table 3).

**Table 3:** Estimated marginal means and SDs of 3-way interaction between region type, reporting rate, and gender for value item 1: “To what extent do you agree or disagree that animals are a valuable part of our society?”

		**Regional**	**Interface**	**Metropolitan**
Male	Low	4.81_a_ (0.41)	4.81_a_ (0.41)	4.45_b_ (1.03)
	High	4.78_a_ (0.47)	4.80_a_ (0.40)	4.79_a_ (0.50)
Female	Low	4.76_b_ (0.53)	4.89_a_ (0.31)	4.89_a_ (0.31)
	High	4.90_a_ (0.30)	4.88_a_ (0.33)	4.86_a_ (0.49)

*Responses scored on a 5-point Likert scale, 1, strongly disagree; 5, strongly agree. SDs in parentheses. Mean comparisons separated by gender. Means that do not share subscripts differ at the p < 0.05 level*.

For value item 5 (*It is not worth the money to take my animal to the vet for general health care when it's not sick or injured*), a small two-way interaction showed participants in the low reporting metro region agreed slightly more than those in the high reporting metro region, hence displaying a slightly less positive attitude toward this item *F*_(2,1800)_ = 6.59, *p* = 0.001, ${\text{ω}}_{\text{p}}^{2}$ = 0.011.

### Attitudes Toward Caring for Animals

This set of items only applied to participants who owned an animal (*n* = 1,114). Gender did not produce significant effects with meaningful effect sizes. A small main effect of region type was found with several items (Table 4). Across these four items, the metropolitan regions consistently displayed the most positive views toward caring for their animals. Interface areas displayed slightly more negative views regarding the perceived behavioral control items.

**Table 4:** Main effects of region type on attitudes toward caring for own animal from two-way factorial ANOVA.

	**Region type main effect ANOVA**					**Estimated marginal means**		
**Item**	***df_IV_***	***df_error_***	***F***	***p***	** ${\text{ω}}_{\text{p}}^{2}$ **	**Regional**	**Interface**	**Metropolitan**
BB1 How my animal feels, whether they are happy and healthy, is important to me	2	1,025	6.67	0.001	0.011	4.82_a_ (0.02)	4.78_a_ (0.02)	4.91_b_ (0.03)
BB3 Providing a high level of care for my animal/s is important to me	2	1,027	8.54	<0.001	0.014	4.74_a_ (0.02)	4.83_b_ (0.02)	4.89_b_ (0.03)
PBC2 I'm unsure how best to care for my animals	2	1,018	6.12	0.002	0.010	1.45_a_ (0.04)	1.60_b_ (0.04)	1.35_a_ (0.06)
PBC3 I can't afford to look after my animals how I would like	2	1,012	8.02	<0.001	0.014	1.49_a_ (0.04)	1.69_b_ (0.05)	1.38_a_ (0.07)

*Items scored on a 5-point Likert scale from (1) strongly disagree to (5) strongly agree. Standard errors in parentheses. Means that do not share subscripts differ at the p < 0.05 level*.

A small effect of report rate was found for the calculated subjective norm variable whereby high reporting regions demonstrated stronger subjective norms (*EMM* = 4.28, *SE* = 0.10) than low reporting areas (*EMM* = 3.65, *SE* = 0.12) *F*_(1,999)_ = 16.31, *p* < 0.001, ${\text{ω}}_{\text{p}}^{2}$ = 0.015.

### Concern for Mistreatment

#### Without Affection Covariate

When the three species were combined into one analysis, the strongest effect found on concern for mistreatment scores was that of gender: females were more concerned (*EMM* = 9.19, *SE* = 0.04) than males (*EMM* = 8.59, *SE* = 0.05), *F*_(1,1623)_ = 92.62, *p* < 0.001, ${\text{ω}}_{\text{p}}^{2}$ = 0.052. This gender effect held true for all species individually, but was stronger for cats (${\text{ω}}_{\text{p}}^{2}$ = 0.078) and horses (${\text{ω}}_{\text{p}}^{2}$ = 0.052) than for dogs (${\text{ω}}_{\text{p}}^{2}$ = 0.026).

A small difference was found between the species whereby dogs elicited more concern (*EMM* = 9.09, *SE* = 0.05) than both cats (*EMM* = 8.84, *SE* = 0.06) and horses (*EMM* = 8.74, *SE* = 0.05), *F*_(2,1623)_ = 11.03, *p* < 0.001, ${\text{ω}}_{\text{p}}^{2}$ = 0.012. Concern for mistreatment of cats and horses did not differ significantly (mean difference = −0.105, *SE* = 0.077, *p* = 0.173).

A small region effect was found for both cats and horses, but not dogs (Table 5). For cats, regional areas displayed less concern for mistreatment than did metropolitan areas (*p* = 0.002) and for horses, metropolitan regions displayed less concern than regional (*p* = 0.01) and interface areas (*p* = 0.004).

**Table 5:** Main effect of region type on mean scale scores of concern for mistreatment items from three-way factorial ANOVA.

	**Region type main effect ANOVA**					**Estimated marginal means**		
**Model**	***df_IV_***	***df_error_***	***F***	***p***	** ${\text{ω}}_{\text{p}}^{2}$ **	**Regional**	**Interface**	**Metropolitan**
Dog	2	551	2.76	0.064	0.006	9.05 (0.09)	9.25 (0.09)	8.96 (0.09)
Cat	2	544	4.99	0.007	0.014	8.60_a_ (0.10)	8.80_ab_ (0.10)	9.06_b_ (0.11)
Horse	2	543	4.89	0.008	0.14	8.83_a_ (0.09)	8.87_a_ (0.09)	8.51_b_ (0.09)
All species	2	1,623	2.73	0.066	0.002	8.83 (0.05)	8.99 (0.05)	8.84 (0.06)

*Responses scored from 0 (not concerned at all) to 10 (extremely concerned). Standard errors in parentheses. Means that do not share subscripts differ at the p < 0.05 level*.

The only statistically significant difference found between high reporting and low reporting areas in concern for mistreatment was when all species were combined in the one analysis. However, the effect size was extremely small *F*_(1,1623)_ = 4.00, *p* = 0.046, ${\text{ω}}_{\text{p}}^{2}$ = 0.002.

#### With Affection Covariate

Repeating the analysis of concern for mistreatment scores with affection as a covariate dramatically improved the overall fit of the statistical models (Table 6).

**Table 6:** Concern for the mistreatment of animals model comparison: adjusted *R*^2^ values for basic model and model with affection included as a covariate (dependent variable = concern for mistreatment scale scores).

	**Model adjusted *R^**2**^* without covariate**	**Model adjusted *R*^**2**^ with covariate**
Dog	0.055	0.241
Cat	0.102	0.199
Horse	0.069	0.201
All species	0.088	0.229

For the combined all species analysis, gender remained the strongest effect (after the affection covariates), but the variance accounted for was reduced from the previous analyses (Table 7). This suggesting that some of the gender effect identified previously was attributable to gender differences in affection.

**Table 7:** All species combined ANCOVA with concern for mistreatment as the dependent variable and affection included as a covariate.

	***df_IV_***	***df_error_***	***F***	***p***	** ${\text{ω}}_{\text{p}}^{2}$ **
Affection cat	1	1,620	34.86	0.000	0.020
Affection dog	1	1,620	69.80	0.000	0.040
Affection horse	1	1,620	49.06	0.000	0.028
Region	2	1,620	1.56	0.211	0.001
Report	1	1,620	0.004	0.948	−0.001
Gender	1	1,620	66.00	0.000	0.038
Species	2	1,620	13.52	0.000	0.015
Region × report	2	1,620	0.74	0.479	0.000
Region × gender	2	1,620	6.00	0.003	0.006
Region × species	4	1,620	5.45	0.000	0.011
Report × gender	1	1,620	1.46	0.228	0.000
Report × species	2	1,620	0.69	0.503	0.000
Gender × species	2	1,620	4.18	0.015	0.004
Region × report × gender	2	1,620	2.13	0.119	0.001
Region × report × species	4	1,620	1.62	0.168	0.001
Region × gender × species	4	1,620	1.17	0.324	0.000
Report × gender × species	2	1,620	0.20	0.820	−0.001
Region × report × gender × species	4	1,620	3.53	0.007	0.006

The species effect remained and increased slightly *F*_(2,1620)_ = 13.52, *p* < 0.001, ${\text{ω}}_{\text{p}}^{2}$ = 0.015. In addition, the region by species effect became slightly stronger *F*_(4, 1620 = 5.45)_, *p* < 0.001, ${\text{ω}}_{\text{p}}^{2}$ = 0.011. In regional areas, people were less concerned about the treatment of cats than dogs (*p* = 0.001). Also, respondents from regional and interface areas were less concerned about cats than respondents in metropolitan areas (regional *p* < 0.001, interface *p* = 0.039). In interface areas, respondents were more concerned about dogs than horses (*p* < 0.001) and cats (*p* = 0.001). Respondents in metropolitan areas cared less about horses than dogs (*p* = 0.001) and cats (*p* < 0.001). Metropolitan respondents also cared less about dogs than respondents in interface areas (*p* = 0.039).

The results of the individual species ANCOVAs on concern about mistreatment scores with affection included as the covariate are summarized in Table 8.

**Table 8:** Summary of three-way factorial ANCOVAs for each species with concern for mistreatment as the dependent variable.

	**Dog**					**Cat**					**Horse**				
	***df_IV_***	***df_error_***	***F***	***p***	** ${\text{ω}}_{\text{p}}^{2}$ **	***df_IV_***	***df_error_***	***F***	***p***	** ${\text{ω}}_{\text{p}}^{2}$ **	***df_IV_***	***df_error_***	***F***	***p***	** ${\text{ω}}_{\text{p}}^{2}$ **
Affection	1	550	136.763	0.000***	0.194	1	543	66.852	0.000***	0.106	1	542	90.523	0.000***	0.139
Region	2	550	2.392	0.092	0.005	2	543	2.918	0.055	0.007	2	542	4.920	0.008**	0.014
Report	1	550	0.599	0.439	−0.001	1	543	1.138	0.287	0.000	1	542	0.289	0.591	−0.001
Gender	1	550	14.852	0.000***	0.024	1	543	30.720	0.000***	0.051	1	542	33.858	0.000***	0.056
Region × report	2	550	4.115	0.017*	0.011	2	543	0.457	0.633	−0.002	2	542	2.512	0.082	0.005
Region × gender	2	550	4.020	0.018*	0.011	2	543	1.609	0.201	0.002	2	542	0.592	0.553	−0.001
Report × gender	1	550	1.667	0.197	0.001	1	543	0.680	0.410	−0.001	1	542	0.125	0.724	−0.002
Region × report × gender	2	550	4.114	0.017*	0.011	2	543	1.750	0.175	0.003	2	542	0.703	0.496	−0.001

*Affection scores for the relevant species used as covariate*.

* *p < 0.05;*

** *p < 0.01*;

*** *p < 0.001*.

**Figure 2:**
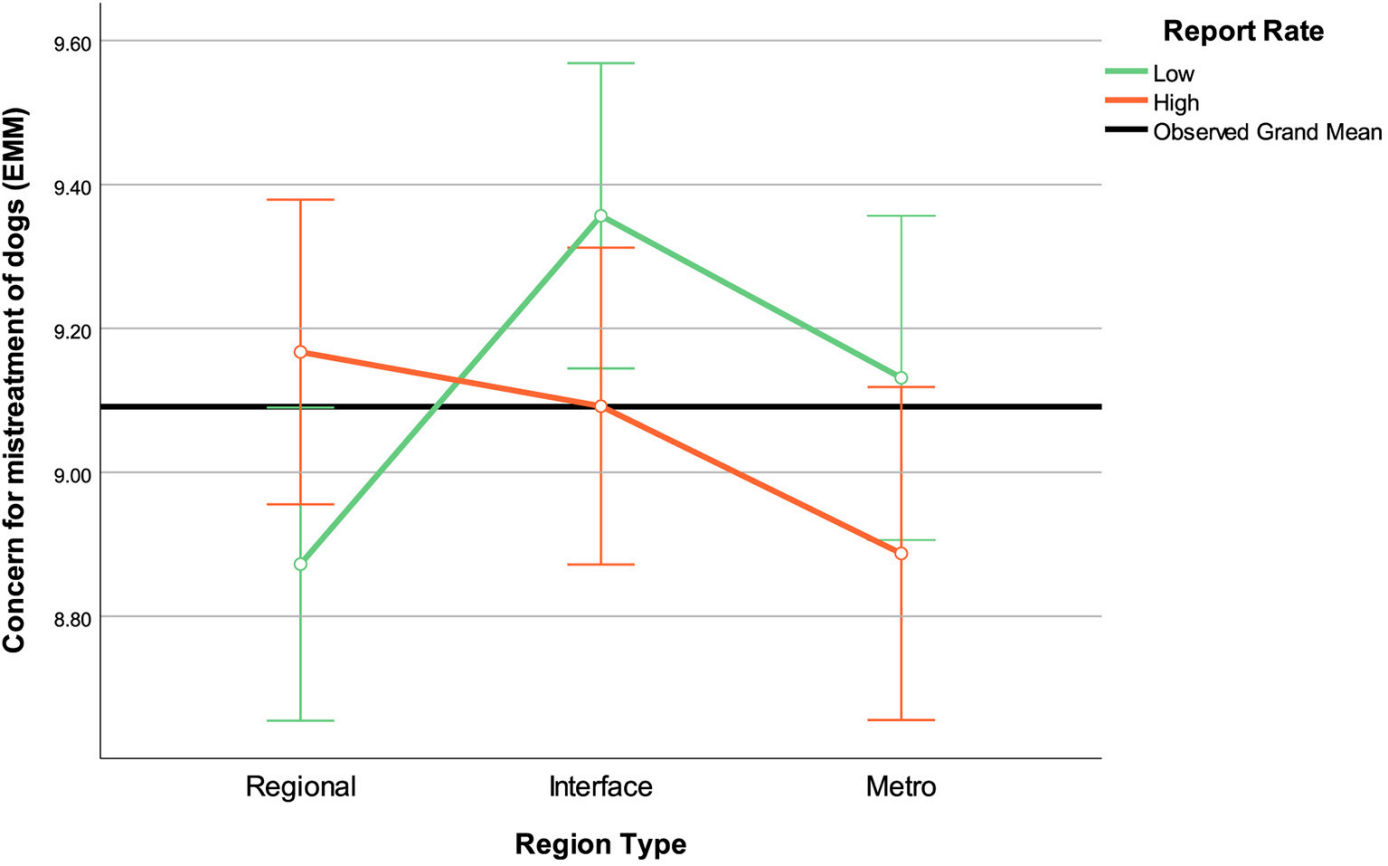
Estimated marginal means of concern for the mistreatment of dogs region by report rate interaction effect. Affection for dogs included in the model as covariate and assessed at the mean = 8.79. Error bars: 95% CI.

For cats, when affection was used as a covariate, the effect of region type decreased and became statistically non-significant. For horses, it remained the same (significant), and for dogs, it remained non-significant. However, for dogs, several interaction effects *became* significant. This included a region by report rate interaction. Simple effect analysis showed that the low reporting regional area had less concern for the mistreatment of dogs than the low reporting interface area, while there were no differences between the regions within the high reporting category (Figure 2).

**Figure 3:**
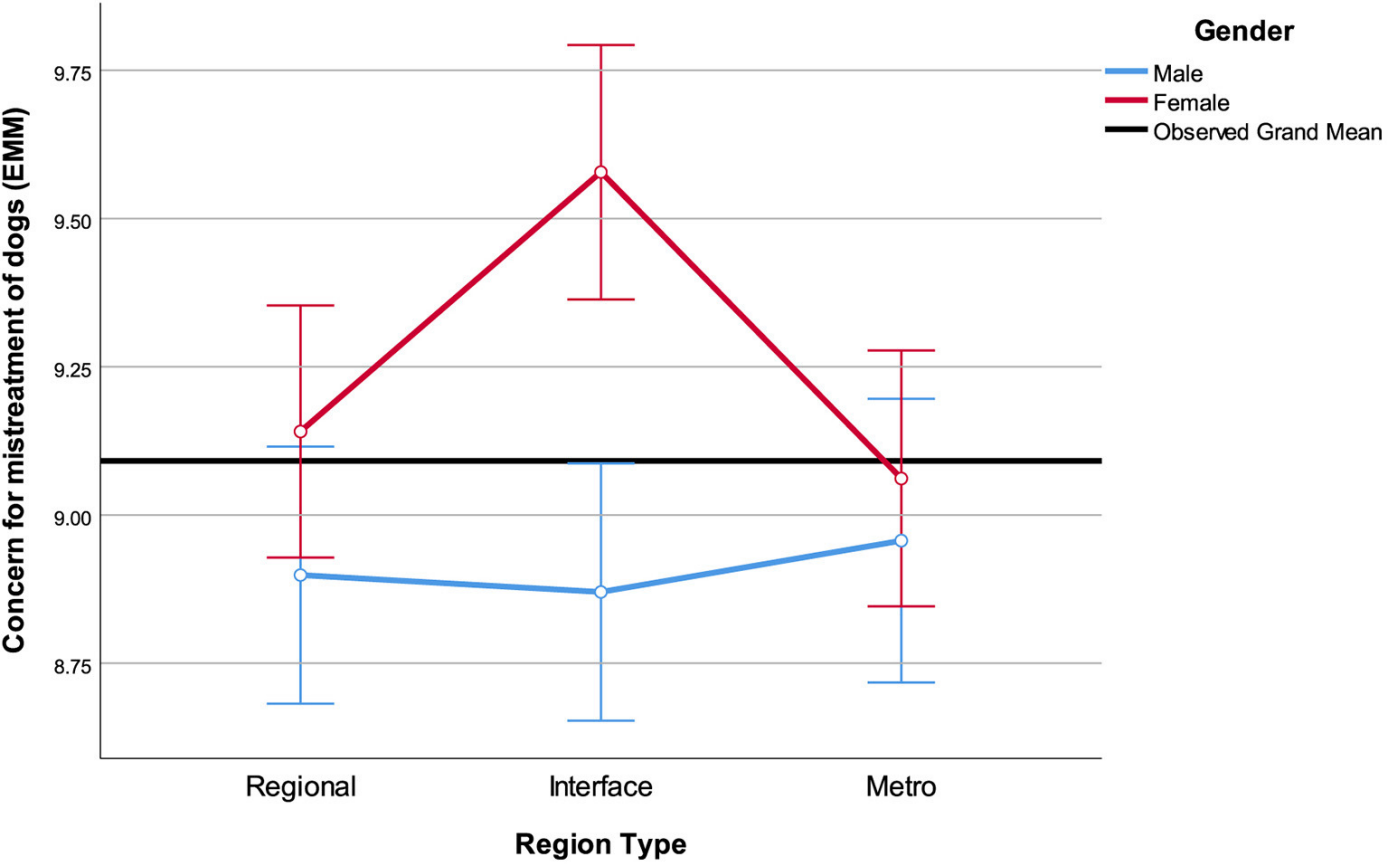
Estimated marginal means of concern for the mistreatment of dogs region by gender interaction effect. Affection for dogs included in the model as covariate and assessed at the mean = 8.79. Error bars: 95% CI.

A region by gender effect on the concern for mistreatment of dogs was also found whereby females in the interface regions had higher levels of concern than all other groups (Figure 3). Males and females in the metropolitan and regional areas did not differ.

**Figure 4:**
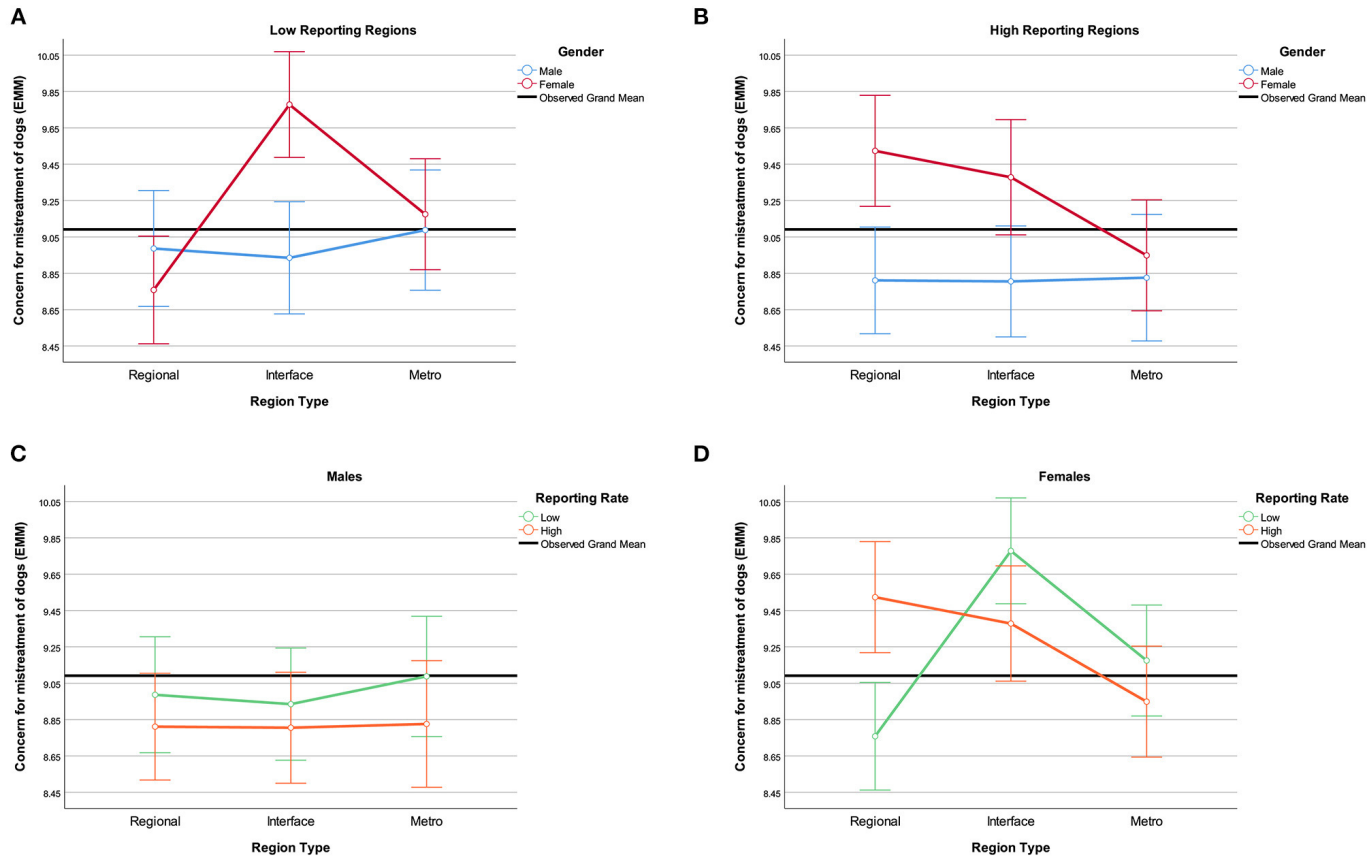
Three-way interaction effect of region type, report rate, and gender on concern for the mistreatment of dogs (estimated marginal means). **(A)** Low reporting regions, **(B)** high reporting regions, **(C)** males, **(D)** females. Affection for dogs covariate evaluated at mean = 8.79. Error bars: 95% CI.

Finally, there was a three-way interaction between region, report rate, and gender for dogs (Figure 4). In low reporting regions (Figure 4A), males and females scored similarly, except in the interface region where females showed significantly more concern than males (*p* < 0.001). In the high reporting regions (Figure 4B), males and females differed in both the regional (*p* = 0.001) and interface regions (*p* = 0.011), but not the metropolitan region. In addition, there was no effect of region type or report rate for males (Figure 4C), but there was for females (Figure 4D). There was no effect of report rate for females in interface or metropolitan regions, but there was in regional areas: females in low reporting regional areas were less concerned than females in high reporting regional areas (*p* < 0.001). Females in the high reporting metropolitan area had less concern than females in the high reporting regional area (*p* = 0.009). Females in the low reporting interface area had more concern than females in the low reporting regional (*p* < 0.001) and metropolitan (*p* = 0.005) areas.

## Discussion

This study aimed to investigate whether regional differences in the reporting and prevalence of animal mistreatment documented in our previous paper (9) were reflected by differences in community attitudes. This was explored through three main research questions, which will now be addressed in turn.

### Do Respondents in Areas of High Reporting Have More Positive Attitudes Toward Animals?

Overall, respondents from high reporting areas had slightly more affection for animals and stronger subjective norms than did those from low reporting areas, but did not differ with respect to valuing animals or their concern for the mistreatment of animals. Given that there were no differences found between high and low reporting regions regarding their concern for different types of animal mistreatment, this suggests that high reporting regions may not have higher standards for animal treatment, but are simply more motivated to report to RSPCA Victoria when they see something wrong. This could be explained in part by their higher levels of affection for animals and stronger perceived social pressures around pet care (subjective norms). It is worth noting, however, that when the investigated species were separated, high reporting regions only showed higher affection levels toward dogs. Given that the majority of reports made to RSPCA Victoria are regarding dogs (31), this is understandable.

That high reporting regions had stronger subjective norms is interesting, given that these items focused on attitudes toward caring for their own animals, not reporting. Subjective norms are personal perceptions of social pressures (11), which are in turn a product of expectations, obligations, and sanctions (45). Perhaps then, the stronger perceived social norms around animal care in high reporting areas may produce a stronger motivation for individuals to enforce consequences for those breaching the accepted standards of care. That is, there is a greater perceived general pressure to provide high standards of care and when they see others not providing that care, they are more likely to “call it out” or report it.

Given the low variance accounted for by the models, there clearly is a range of other factors, not accounted for here, that are involved in why different regions display different reporting rates despite having a similar prevalence of mistreatment. An alternative explanation could be that high reporting areas are more likely to report as a result of better relationships with RSPCA or authorities in general, whereas people in low reporting areas may take alternative actions to address the situation. In reality, it is likely to be a complex combination of many factors, including those small but significant factors identified here, which together summate to a significant difference in outcomes at the population level. So while our hypothesis is somewhat supported by the data, there are likely to be many additional contributing factors. However, interventions to improve reporting could still benefit from addressing those factors found to differ here (affection and social norms). Such an approach would demonstrate a causal explanation of reporting rates.

### Do Respondents in Areas of High Prevalence Have More Negative Attitudes Toward Animals?

Participants in areas of high prevalence (regional areas) did not display lower levels of affection or concern for the mistreatment of animals as a whole, nor did they value animals less. However, differences were found for several attitude items regarding caring for their own animals; two behavioral beliefs and two control beliefs. In addition, differences were found between the regions regarding the levels of concern for mistreatment when broken down into the different species; however, these will be discussed in the species section below (section Do Attitudes Differ for Different Target Species?).

Regarding attitudes toward caring for their own pets, the only item for which regional (higher prevalence areas) scored lower than *both* the other regions (thus supporting the hypothesis) was for behavioral belief 3 (BB3 *Providing a high level of care for my animal/s is important to me*). For all other items with significant differences (BB1 *How my animal feels, whether they are happy and healthy, is important to me*, PBC2 *I'm unsure how best to care for my animals*, PBC3 *I can't afford to look after my animals how I would like*), the regional areas scored similarly to one other region type, with the third region type accounting for the significant difference. The differences in sentiment of these items suggest that while caring for their animals is important to them (BB1 and BB2 *My animal's health and happiness depend on how I look after them*), providing a “high level of care” may be not as much (BB3). Given that the regional areas were the most socioeconomically disadvantaged regions sampled, the most intuitive explanation is that they have more conflicting priorities or consider high levels of care unattainable due to resource constraints. However, regional respondents did not score highest (more strongly agree) on the item related to financial difficulties (PBC3); indeed, the interface regions did. As such, given the generally more politically conservative nature of regional areas (46), perhaps their response to BB3 reflects a more pragmatic view and that “high” levels of care are unnecessary. Indeed, Kellert (47) identified significant regional differences in attitudes toward animals in the USA, with the Southern areas, which are typically more conservative, having less concern for animals and a more utilitarian view. Furthermore, the same study found that people who have completed less education, are older, live in cities of < 1 million, and are farmers have more utilitarian views toward animals, being primarily concerned with their practical and material value (47). In the more extreme sense, Dhont and Hodson (48) found that “right-wing” adherents had more favorable views toward animal exploitation and identified this as partly a function of their belief in human superiority over animals. These findings are also reflected in Clark et al. (21). More investigation, including detailed qualitative methods, is required to explore this further. Additional factors, such as how the animal was acquired, are also likely to be important (27).

### Do Attitudes Differ for Different Target Species?

Our results demonstrate that people's attitudes and affection differ between species with clear biases toward dogs. This is consistent with a substantial body of research documenting species preferences, with dogs often being favored over cats (30, 49) and being more highly regarded than other species in terms of various cognitive and emotional capacities (50–52). The present results also demonstrate that views about cats are more polarized than dogs and to a lesser extent, horses, with a significant proportion of people not liking cats, even “hating” them. Indeed, Toukhsati et al. (40) found similar results comparing cats and dogs. Yet, this current study is the first that we are aware of to incorporate horses in such comparisons.

These polarized views toward cats are clearly reflected in the media, online, and to some extent, literature. On one hand, the “Internet Cat Phenomenon” has seen cat-related media attracting billions of views (53) sparking such proclamations as “cats win the internet” (54). On the other hand, articles in popular media streams often reflect common beliefs that cats are “selfish, unfeeling, environmentally devastating creatures” (55). Wildlife predation and nuisance behaviors such as fighting, spraying, and defecating in gardens are common complaints against cats (56–58) while perceptions of cat behavior as aloof and independent are also likely to contribute to negative attitudes (59). Such species preferences have also been linked to gender and personality traits (60).

Importantly, our results also demonstrate that even when differences in affection for these species are taken into account, species differences in people's concern for their mistreatment remain. People were more concerned about the mistreatment of dogs, than horses or cats. This suggests that not only do people have differing levels of affection for different species, but that there are also differing standards for their treatment, independent of affection. This is likely related to a range of factors such as utility, the role of the species, perceived needs of the animals, and obligations (61).

Interestingly, the species differences in affection levels are directly reflected in the number of reports made to RSPCA. That is, dogs are most commonly reported, followed by horses, and lastly cats (31). While the lesser number of horse-related reports can be attributed to the fact that there are fewer horses in Victoria than dogs, the mistreatment of cats is reported less than half as often as dogs, despite similar levels of ownership (62). While this could also be a result of cats being less visible than dogs and horses in society (i.e., they are kept indoors out of sight or roaming/scarce), it is likely that the mistreatment of cats is underrepresented in official reports due to differences in both their accepted level of treatment and the affection they garner.

Interesting interactions between region type and species were also found. Metropolitan respondents were generally more sympathetic and positive toward cats and less so toward horses. In contrast, regional respondents disliked cats more and were less concerned about their treatment, although their reduced concern was likely attributable to the reduced affection given that the effect disappeared when affection was incorporated as a covariate. These regional differences are consistent with lifestyle trends and the types of pets able to be kept in those areas. That metropolitan respondents were less concerned about horses may be due to a lack of familiarity with the species and what is appropriate care as they are the least likely to have the space to own or be closely involved with horses (unless agisting elsewhere). There is also anecdotal evidence to suggest that attitudinal trends may differ between countries. In the USA, urban residents seem to have greater concern than rural residents for other horse welfare issues such as the culling of wild horses (63), while the opposite has been observed in Australia, i.e., rural residents are more concerned (64). While regional differences in attitudes toward cats have not previously been studied *per se*, Hall et al. (56) identified differences in cat ownership practices in Sydney, Australia (a densely populated capital city, population=5.3 million) compared with Wollongong (a smaller Australian coastal city, population = 295,000). Survey participants from Sydney were more likely to keep their cats solely indoors, while Wollongong participants mostly allowed their cats indoors and outdoors. This trend was also identified in Ohio, USA whereby cat owners in rural areas were more likely to allow their cats to roam freely and were less likely to have them desexed than those in urban areas (65). Such differences in practices could suggest different cultural relationships with cats; in more regional areas, cats are kept in a more independent way and relationships are not as intense as those in urban areas where cats are often kept indoors and interactions are frequent. However, feeding roaming cats (semi-ownership) is also common in urban areas, with semi-owners often having strong connections with roaming cats (40, 66). In reality, there is likely to be a range of factors pre-disposing metropolitan residents to greater affection and concern for cats than regional residents. As such, species-specific intervention messaging is recommended, particularly improving attitudes toward cats in regional areas.

### Complex Interactions

While a number of higher order interaction effects between region type, reporting rates, gender, and target species were identified, it is not appropriate to generalize from these smaller subsamples. Any explanation of these effects would be pure speculation, and hence will not be attempted. However, it is important to note such differences as they highlight unexpected idiosyncrasies that need to be investigated and understood when developing intervention strategies.

### Gender

Significant differences in attitudes were often found between genders. On average, females had higher levels of affection for animals, valued animals more, and were more concerned for the mistreatment of animals, particularly cats and horses. That females have more positive attitudes toward animals is a well-recognized phenomenon, typically with a moderate effect size [see (67) for review]. Females are also more likely to label themselves as “cat people” than males, reflecting long-standing cultural stereotypes (60). However, as Herzog (67) stresses, there is considerable overlap of distributions between the genders and often the variability *between* genders is less than the variability *within* genders. In addition to differences in the strength or direction of attitudes, Henry (34) identified *structural* differences between genders in their attitudes toward the treatment of animals, proposing that females had a broader recognition of what constituted cruelty than males.

Given the existing evidence for gender differences in attitudes, it is important to note that gender did not have a significant effect on the attitudes toward caring for one's own animal. This is an important distinction for designers of intervention strategies, particularly when identifying target audiences relative to the intervention's aims. For example, if the aim is to increase community concern for animal mistreatment, then targeting males differently or more specifically may be appropriate. However, if the goal is to improve people's attitudes toward caring for their own animals, all genders would be equally appropriate targets.

It is important to recognize that the views of non-binary individuals were not adequately represented among the final sample to incorporate non-binary as a gender category in the analyses (*n* = 2). To investigate the views of non-binary individuals, either a much larger random sample or targeted recruitment would be required to capture a robust number of individuals in this group.

### Additional Limitations

While this was a random, representative survey, being voluntary, there is still the potential for bias toward people who are more concerned about or engaged with animal-related issues. That is, people who are more interested in animals may be more likely to participate and those who are not may be more likely to decline. Consequently, the sample may not fully capture individuals who do not care about animals and as such, potentially those who do not treat their animals appropriately for this reason.

There was a heavy skew in the concern for mistreatment items suggesting an element of social desirability bias in the responses. The items were intended to cover a range of issues, from those that may seem fairly innocuous (the animal being on its own and receiving little attention) through to overt cruelty (someone intentionally hurting the animal). Pre-testing confirmed that people responded differently to these items, although in the final survey, many scored them similarly—being “extremely concerned” for all items. This is likely a result of social desirability and self-selection bias toward people who were overly concerned about animals. In addition, the order of the items could have had an effect. The less serious items were delivered first and if the participant rated them highly, then by the time they got to the more serious items, they may not have had higher ratings to give. This could be improved by reversing the order of the items in future studies. However, despite the reduced variability, significant effects were still able to be found, reflecting the strength of the effect.

The survey was only delivered in English and therefore had the potential for sampling bias. However, only 0.8% of the total number of people contacted were excluded as they did not speak English.

### Implications for Intervention Strategies

One of the underlying impetuses for community attitudinal research is to understand factors underlying a social issue that can be targeted through some sort of intervention. The inconsistencies of results found in this study highlight the need for interventions to have clear targets with regards to a range of factors including the audience, the behavior, and the relevant species. Interventionists should target smaller regions and thoroughly investigate their unique perspectives, challenges, and strengths. While the current study was quantitative in nature, qualitative research and community outreach should be included at the next stage of intervention development to achieve this in-depth understanding. Co-design and pre-testing of interventions is also vital to ensure they are relevant, appropriate, and acceptable to the target audience (3). As community attitudes are complex with many contributing factors, it should be expected that targeting significant attitudinal differences may only have small impacts at the community level and take time for positive outcomes to be seen. It is also important to note that positive outcomes may be counter-intuitive, for example, there may be an increase in mistreatment report rates because people are more aware and concerned, not because prevalence has increased. Finally, due to the complex nature of these issues, campaigns aimed at changing attitudes should also be complemented with other resource- and support-based measures for a more holistic and effective approach.

## Data Availability Statement

The raw data supporting the conclusions of this article will be made available by the authors, without undue reservation.

## Ethics Statement

The studies involving human participants were reviewed and approved by University of Melbourne Veterinary and Agricultural Sciences Human Ethics Advisory Group. Written informed consent for participation was not required for this study in accordance with the national legislation and the institutional requirements.

## Author Contributions

CG, JF, and GC: conceptualization and methodology. CG and GC: formal analysis. CG: writing—original draft preparation. JF, RC, and GC: writing—review and editing. CG, JF, and RC: project administration and funding acquisition. All authors contributed to the article and approved the submitted version.

## Funding

Contracting of the Social Research Center for data collection was funded by RSPCA Victoria predominantly through a grant received from the Victorian State Government's Department of Economic Development, Jobs, Transport and Resources (now Department of Jobs, Precincts and Regions).

## Conflict of Interest

The authors declare that the research was conducted in the absence of any commercial or financial relationships that could be construed as a potential conflict of interest.

## Publisher's Note

All claims expressed in this article are solely those of the authors and do not necessarily represent those of their affiliated organizations, or those of the publisher, the editors and the reviewers. Any product that may be evaluated in this article, or claim that may be made by its manufacturer, is not guaranteed or endorsed by the publisher.

## References

[1] Sonntag Q Overall KL. Key determinants of dog and cat welfare: behaviour, breeding and household lifestyle. Revue Sci Tech. (2014) 33:213–20. 10.20506/rst.33.1.2270 25000794

[2] McMillan FD Duffy DL Zawistowski SL Serpell JA. Behavioral and psychological characteristics of canine victims of abuse. J Appl Anim Welfare Sci. (2015) 18:92–111. 10.1080/10888705.2014.962230 25257564

[3] Glanville C Abraham C Coleman G. Human behaviour change interventions in animal care and interactive settings: a review and framework for design and evaluation. Animals. (2020) 10:2333. 10.3390/ani10122333 33302506 PMC7764651

[4] Philpotts I Dillon J Rooney N. Improving the welfare of companion dogs-is owner education the solution? Animals. (2019) 9:662. 10.3390/ani9090662 31500203 PMC6770859

[5] Wood S. Animal Cruelty Hotspots. Fairfax Media Publications Pty Limited (2018). Available online at: https://www.colliemail.com.au/story/4694701/animal-cruelty-hot-spot/ (accessed July 30, 2019).

[6] Mackander M. Maroochydore and Nambour Coast's Worst for Animal Cruelty. APN Newspapers Pty Ltd (2015). Available online at: https://www.sunshinecoastdaily.com.au/news/lack-of-action-cause-of-abuse/2503676/ (accessed July 30, 2019).

[7] Kirkham R. Hepburn Shire Records Most Reports of Animal Cruelty Per Capita in State. Fairfax Media Publications Pty Limited (2018). Available online at: https://www.thecourier.com.au/story/5778576/hepburn-shire-records-most-reports-of-animal-cruelty-per-capita-in-state/ (accessed July 30, 2019).

[8] Lavigueur N. West Yorkshire Revealed as Animal Cruelty Hotspot as RSPCA Convictions Surge. (2012). Available online at: https://www.examinerlive.co.uk/news/west-yorkshire-news/west-yorkshire-revealed-animal-cruelty-4955095 (accessed September 11, 2020).

[9] Glanville C Ford J Coleman G. Animal cruelty and neglect: prevalence and community actions in Victoria, Australia. Animals. (2019) 9:1121. 10.3390/ani9121121 31835849 PMC6940924

[10] Victorian State Government. Victorian Local Government Comparator Groups. (2015). Available online at: https://knowyourcouncil.vic.gov.au/__data/assets/pdf_file/0009/29439/DOC-15-313642-Victorian-Local-Government-Comparator-Groups-2015-FINAL.pdf (accessed April 11, 2021).

[11] Ajzen I. From intentions to actions: a theory of planned behavior. In: Kuhl J Beckmann J editors. Action Control: From Cognition to Behavior. Berlin; Heidelberg: Springer (1985). 11–39. 10.1007/978-3-642-69746-3_2

[12] Ajzen I. The theory of planned behavior. Organ Behav Hum Decis Process. (1991) 50:179–211. 10.1016/0749-5978(91)90020-T

[13] Ajzen I Fishbein M. The influence of attitudes on behavior. In: Albarracín D. Johnson BT Zanna MP editors. The Handbook of Attitudes. Mahwah, NJ: Lawrence Erlbaum Associates Publishers (2005). 173–221.

[14] Reyna C Brandt M Viki GT. Blame it on hip-hop: anti-rap attitudes as a proxy for prejudice. Group Process Intergroup Relat. (2009) 12:361–80. 10.1177/1368430209102848

[15] Tucci J Mitchell J Goddard C. Doing Nothing Hurts Children: Community Attitudes About Child Abuse and Child Protection in Australia. Australian Childhood Foundation (2010). Available online at: https://professionals.childhood.org.au/app/uploads/2018/07/Research20Doing20Nothing20Hurts20Children202010.pdf (accessed October 14, 2020).

[16] Webster K Ward A Diemer K Flood M Powell A Forster K. Attitudinal support for violence against women: what a population-level survey of the Australian community can and cannot tell us. Australian J Soc Issues. (2019) 54:52–75. 10.1002/ajs4.56

[17] Morton R Hebart ML Whittaker AL. Explaining the gap between the ambitious goals and practical reality of animal welfare law enforcement: a review of the enforcement gap in Australia. Animals. (2020) 10:482. 10.3390/ani10030482 32183062 PMC7142490

[18] Coleman GJ Rohlf V Toukhsati SR Blache D. Public attitudes predict community behaviours relevant to the pork industry. Anim Product Sci. (2018) 58:416–23. 10.1071/AN16776

[19] Bray HJ Buddle EA Ankeny RA. What are they thinking? Consumer attitudes to meat production in Australia. Anim Product Sci. (2017) 57:2345–52. 10.1071/AN17361

[20] Rice M Hemsworth LM Hemsworth PH Coleman GJ. The impact of a negative media event on public attitudes towards animal welfare in the red meat industry. Animals. (2020) 10:619. 10.3390/ani10040619 32260202 PMC7222821

[21] Clark B Stewart GB Panzone LA Kyriazakis I Frewer LJ. A systematic review of public attitudes, perceptions and behaviours towards production diseases associated with farm animal welfare. J Agric Environ Ethics. (2016) 29:455–78. 10.1007/s10806-016-9615-x

[22] Gates MC Walker J Zito S Dale A. A survey of opinions towards dog and cat management policy issues in New Zealand. N Z Vet J. (2019) 67:315–22. 10.1080/00480169.2019.1645627 31319780

[23] Acutt D Signal T Taylor N. Mandated reporting of suspected animal harm by australian veterinarians: community attitudes. Anthrozoos. (2015) 28:437–47. 10.1080/08927936.2015.1052276

[24] Rohlf VI Bennett PC Toukhsati S Coleman G. Why do even committed dog owners fail to comply with some responsible ownership practices? Anthrozoös. (2010) 23:143–55. 10.2752/175303710X12682332909972

[25] Rohlf VI Bennett PC Toukhsati S Coleman G. Beliefs underlying dog owners' health care behaviors: results from a large, self-selected, internet sample. Anthrozoös. (2012) 25:171–85. 10.2752/175303712X.13316289505341

[26] Elliott A Howell TJ McLeod EM Bennett PC. Perceptions of responsible cat ownership behaviors among a convenience sample of Australians. Animals. (2019) 9:703. 10.3390/ani9090703 31546938 PMC6769723

[27] Freiwald A Litster A Weng H.-Y. Survey to investigate pet ownership and attitudes to pet care in metropolitan Chicago dog and/or cat owners. Prevent Vet Med. (2014) 115:198–204. 10.1016/j.prevetmed.2014.03.025 24774476

[28] Henry B. The relationship between animal cruelty, delinquency, and attitudes toward the treatment of animals. Soc Anim. (2004) 12:185–207. 10.1163/1568530042880677

[29] Hawkins RD Williams JM. Children's attitudes towards animal cruelty: exploration of predictors and socio-demographic variations. Psychol Crime Law. (2020) 26:226–47. 10.1080/1068316X.2019.1652747

[30] Selby LA Rhoades JD. Attitudes of the public towards dogs and cats as companion animals. J Small Anim Pract. (1981) 22:129–37. 10.1111/j.1748-5827.1981.tb00592.x 7230750

[31] RSPCA Victoria. Financial Report 2018/2019. (2019). Available online at: https://www.rspcavic.org/documents/About%20us/Annual%20Report/2019/RSPCA%20Financial%20Report%20YE%2030%20June%202019%20-%20FINAL.pdf (accessed September 14, 2020).

[32] Batt S. Human attitudes towards animals in relation to species similarity to humans: a multivariate approach. Biosci Horizons. (2009) 2:180–90. 10.1093/biohorizons/hzp021

[33] Borgi M Cirulli F. Attitudes toward animals among kindergarten children: species preferences. Anthrozoös. (2015) 28:45–59. 10.2752/089279315X14129350721939

[34] Henry B. Can attitudes about animal neglect be differentiated from attitudes about animal abuse? Soc Anim. (2009) 17, 21–37. 10.1163/156853009X393747

[35] Khor MM Davey G Zhao X. Why do people feed free-roaming cats? The role of anticipated regret in an extended theory of planned behavior in Malaysia. Anthrozoös. (2018) 31:101–16. 10.1080/08927936.2018.1406204

[36] Kauppinen T Valros A Vesala KM. Attitudes of dairy farmers toward cow welfare in relation to housing, management and productivity. Anthrozoös. (2013) 26:405–20. 10.2752/175303713X13697429463718

[37] Lafollette MR Cloutier S Brady C Gaskill BN O'haire ME. Laboratory animal welfare and human attitudes: a cross-sectional survey on heterospecific play or “rat tickling”. PLoS ONE. (2019) 14:e0220580. 10.1371/journal.pone.0220580 31412066 PMC6693744

[38] Borges JAR Domingues CHDF Caldara FR Rosa NPD Senger I Guidolin DGF. Identifying the factors impacting on farmers' intention to adopt animal friendly practices. Prevent Vet Med. (2019) 170:104718. 10.1016/j.prevetmed.2019.104718 31421489

[39] Boateng GO Neilands TB Frongillo EA Melgar-Quiñonez HR Young SL. Best practices for developing and validating scales for health, social, and behavioral research: a primer. Front Public Health. (2018) 6:149. 10.3389/fpubh.2018.00149 29942800 PMC6004510

[40] Toukhsati SR Bennett PC Coleman GJ. Behaviors and attitudes towards semi-owned cats. Anthrozoös. (2007) 20:131–42. 10.2752/175303707X207927

[41] American Association for Public Opinion Research. Standard Definitions: Final Dispositions of Case Codes Outcome Rates for Surveys. AAPOR (2016). Available online at: https://www.aapor.org/AAPOR_Main/media/publications/Standard-Definitions20169theditionfinal.pdf (accessed October 8, 2019).

[42] Taylor N Signal TD. Empathy and attitudes to animals. Anthrozoös. (2005) 18:18–27. 10.2752/089279305785594342

[43] Hays WL. Statistics. Fort Worth, TX: Holt, Rinehart and Winston Inc. (1988).

[44] Ajzen I Driver BL. Prediction of leisure participation from behavioral, normative, and control beliefs: an application of the theory of planned behavior. Leisure Sci. (1991) 13:185–204. 10.1080/0149040910951313715885041

[45] Schwartz SH. Normative influences on altruism. In: Berkowitz L editor. Advances in Experimental Social Psychology. New York, NY: Academic Press (1977). 221–279. 10.1016/S0065-2601(08)60358-5

[46] Victorian Electoral Commission. State Election Results Map. (2018). Available online at: https://www.vec.vic.gov.au/images/maps/Map-stateDistrictResults2018.png (accessed June 7, 2020).

[47] Kellert SR. American attitudes toward knowledge of animals: an update. In: Fox MW Mickley LD editors. Advances in Animal Welfare Science. Washington, DC: The Humane Society of the United States (1984). p 177–213.

[48] Dhont K Hodson G. Why do right-wing adherents engage in more animal exploitation and meat consumption? Pers Individ Diff. (2014) 64:12–7. 10.1016/j.paid.2014.02.002

[49] Travaglia M Miller KK. Cats in the Australian environment: what's your purr-spective? Australasian J Environ Manage. (2018) 25:153–73. 10.1080/14486563.2017.1369465

[50] Howell TJ Toukhsati S Conduit R Bennett P. The perceptions of dog intelligence and cognitive skills (PoDIaCS) survey. J Vet Behav. (2013) 8:418–24. 10.1016/j.jveb.2013.05.005

[51] Wilkins AM McCrae LS McBride EA. Factors affecting the human attribution of emotions toward animals. Anthrozoös. (2015) 28, 357–369. 10.1080/08927936.2015.1052270

[52] Davis SL Cheeke PR. Do domestic animals have minds and the ability to think? A provisional sample of opinions on the question. J Anim Sci. (1998) 76:2072–9. 10.2527/1998.7682072x 9734856

[53] Myrick JG. Emotion regulation, procrastination, and watching cat videos online: who watches Internet cats, why, and to what effect? Comput Hum Behav. (2015) 52:168–76. 10.1016/j.chb.2015.06.001

[54] Tucker A. How Cats Evolved to Win the Internet. New York Times (2016). Available online at: https://www.nytimes.com/2016/10/16/opinion/sunday/how-cats-evolved-to-win-the-internet.html (accessed June 7, 2020).

[55] Stromberg J. What Research Says About Cats: They're Selfish, Unfeeling, Environmentally Harmful Creatures. Vox (2014). Available online at: https://www.vox.com/2014/10/16/6982177/the-case-against-owning-cats (accessed June 7, 2020).

[56] Hall CM Adams NA Bradley JS Bryant KA Davis AA Dickman CR. Community attitudes and practices of urban residents regarding predation by pet cats on wildlife: an international comparison. PLoS ONE. (2016) 11:e0151962. 10.1371/journal.pone.0151962 27050447 PMC4822884

[57] Grayson J Calver M Styles I. Attitudes of suburban Western Australians to proposed cat control legislation. Austral Vet J. (2002) 80:536–43. 10.1111/j.1751-0813.2002.tb11030.x 12398314

[58] Toukhsati SR Young E Bennett PC Coleman GJ. Wandering cats: attitudes and behaviors towards cat containment in Australia. Anthrozoös. (2012) 25:61–74. 10.2752/175303712X13240472427195

[59] Grigg EK Kogan LR. Owners' attitudes, knowledge, and care practices: exploring the implications for domestic cat behavior and welfare in the home. Animals. (2019) 9:978. 10.3390/ani9110978 31731680 PMC6912669

[60] Perrine RM Osbourne HL. Personality Characteristics of Dog and Cat Persons. Anthrozoös. (1998) 11:33–40. 10.1080/08927936.1998.11425085

[61] Serpell JA. Factors influencing human attitudes to animals and their welfare. Anim Welfare. (2004) 13:S145–52.

[62] Animal Medicines Australia. Pets in Australia: A National Survey of Pets and People. (2019). Available: https://animalmedicinesaustralia.org.au/wp-content/uploads/2019/10/ANIM001-Pet-Survey-Report19_v1.7_WEB_high-res.pdf (accessed August 31, 2020).

[63] Lokting B. The Wild Horse Wars. The Washington Post Magazine (2020). Available online at: https://www.washingtonpost.com/magazine/2020/11/18/wild-horses-ranchers-animal-rights-activists/ (accessed April 21, 2021).

[64] Albeck-Ripka L. Majestic Icon or Invasive Pest? A War Over Australia's Wild Horses. The New York Times (2020). Available online at: https://www.nytimes.com/2020/06/28/world/australia/brumbies-horses-culling.html (accessed April 21, 2021).

[65] Lord LK. Attitudes toward and perceptions of free-roaming cats among individuals living in Ohio. J Am Vet Med Assoc. (2008) 232:1159–67. 10.2460/javma.232.8.1159 18412526

[66] Finkler H Terkel J. Dichotomy in the emotional approaches of caretakers of free-roaming cats in urban feeding groups: findings from in-depth interviews. Anthrozoös. (2011) 24:203–18. 10.2752/175303711X12998632257413

[67] Herzog HA. Gender differences in human-animal interactions: a review. Anthrozoös. (2007) 20:7–21. 10.2752/089279307780216687

